# *LcMYB4*, an unknown function transcription factor gene from sheepgrass, as a positive regulator of chilling and freezing tolerance in transgenic Arabidopsis

**DOI:** 10.1186/s12870-020-02427-y

**Published:** 2020-05-27

**Authors:** Xiaoxia Li, Junting Jia, Pincang Zhao, Xiufang Guo, Shuangyan Chen, Dongmei Qi, Liqin Cheng, Gongshe Liu

**Affiliations:** 1 Key Laboratory of Plant Resources, Institute of Botany, The Chinese Academy of Sciences, Beijing, China; 2 Guangdong Provincial Key Laboratory for Crop Germplasm Resources Preservation and Utilization, Agro-biological Gene Research Center, Guangdong Academy of Agricultural Sciences, Guangzhou, China; 3 College of management science and engineering, Hebei University of Economics and Business, Shijiazhuang, China

**Keywords:** Sheepgrass, MYB transcriptional factor, Freezing tolerance, Chilling stress, Transgenic

## Abstract

**Background:**

Sheepgrass (*Leymus chinensis* (Trin.) Tzvel) is a perennial forage grass that can survive extreme freezing winters (− 47.5 °C) in China. In this study, we isolated an unknown function MYB transcription factor gene, *LcMYB4*, from sheepgrass*.* However, the function of *LcMYB4* and its homologous genes has not been studied in other plants.

**Results:**

The expression of the *LcMYB4* gene was upregulated in response to cold induction, and the LcMYB4 fusion protein was localized in the nucleus, with transcriptional activation activity. Biological function analysis showed that compared with WT plants, *LcMYB4*-overexpressing Arabidopsis presented significantly increased chilling and freezing tolerance as evidenced by increased germination rate, survival rate, and seed setting rate under conditions of low temperature stress. Furthermore, *LcMYB4*-overexpressing plants showed increased soluble sugar content, leaf chlorophyll content and superoxide dismutase activity but decreased malondialdehyde (MDA) under chilling stress. Moreover, the expression of the *CBF1*, *KIN1*, *KIN2* and *RCI2A* genes were significantly upregulated in transgenic plants with chilling treatment. These results suggest that *LcMYB4* overexpression increased the soluble sugar content and cold-inducible gene expression and alleviated oxidative damage and membrane damage, resulting in enhanced cold resistance in transgenic plants. Interestingly, our results showed that the LcMYB4 protein interacts with fructose-1,6-bisphosphate aldolase protein1 (LcFBA1) and that the expression of the *LcFBA1* gene was also upregulated during cold induction in sheepgrass, similar to *LcMYB4*.

**Conclusion:**

Our findings suggest that *LcMYB4* encodes MYB transcription factor that plays a positive regulatory role in cold stress.

## Background

Chilling (low temperatures above 0 °C) and freezing can adversely affect plant growth and reduce crop yield [1]. Under cold conditions, gene expression, enzyme activity, metabolism and cellular architecture are changed [2, 3], and many transcription factor (TF) genes are involved in the response and tolerance to cold stresses in plants [4–7]. Expression profiling of the model plant Arabidopsis treated with cold revealed that up to 20% of genes in the genome are regulated by cold [8]. The explicit mechanism of cold stress-induced transcription in plants is the C-repeat (CRT)-binding factor and/or dehydration-responsive element (DRE) binding factor (CBF/DREB) cascade [9]. However, the expression levels of CBFs determine the expression of cold-responsive (*COR*) gene, which can encode cryoprotective proteins to enhance the freezing resistance of plant [7, 10, 11]. Previous studies have shown that the MYB15 can bind to the specific promoter elements of *CBF* and inhibit the expression of *CBFs*, while the *CBFs* can be induced by ICE1 (Inducer of CBF expression 1) and ICE1-like transcription factors [12–14]. The MYB family is one of the most important transcription factor (TF) families studied in plants [15]. The first plant MYB gene to be isolated, COLORED1 (C1), is related to anthocyanin synthesis in maize (*Zea mays*) kernels [16]. In contrast to animals, in plants, MYB TFs are involved in various developmental processes and many complex physiological processes, including regulating plant morphogenesis, growth and development, participating in plant stress response to biotic and abiotic stresses, and regulating plant primary and secondary metabolism processes [17–20]. MYB proteins contain highly conserved MYB DNA-binding domains, which can be divided into the following four types: MYB1R, R2R3-MYB, R1R2R3 MYB (MYB3R), and 4RMYB, based on the repeats of the MYB DNA-binding domains (one, two, three, and four), respectively [17].

Previous studies have shown that MYB TF family genes are involved in biotic and abiotic stress through the regulation of gene expression [5, 18, 21–23]. In *Arabidopsis thaliana*, AtMYB30, AtMYB60, and AtMYB96 are R2R3-MYB-type proteins and are involved in responses to drought stress and disease resistance [17]. However, AtMYB15 responds to cold and drought stress, and AtMYB108 is required for biotic and abiotic stress responses [24]. Studies on the functions of MYB proteins in rice (*Oryza sativa*) indicated that the overexpression of *OsMYB3R-2*, which is an R1R2R3 MYB gene, can increase tolerance to freezing, drought, and salt stress in transgenic Arabidopsis [23]. The *OsMYBS3* gene can adapt short- and long-term cold stress and is a novel MYBS3-dependent pathway that confers cold tolerance in rice [25], and the *OsMYB2* (R2R3-type MYB) gene enhances the salt, cold, and dehydration tolerance in rice [5]. Recent studies on *Cichorium intybus* have found that the R2R3-MYB TFs *CiMYB5* and *CiMYB3* regulate fructan 1-exohydrolase expression in response to abiotic stress and hormonal cues [21].

Sheepgrass (*Leymus chinensis* (Trin.) Tzvel) is a cold-season forage grass widely distributed on the eastern Eurasian steppe [26, 27], and the total area of sheepgrass grasslands in China is approximately 220,000 km^2^ [28]. Previous studies have showed that sheepgrass can grows well in drought condition(< 6% soil moisture), high salt stress (600 mM NaCl), and extreme freezing winters (− 47.5 °C) [28–30]. We identified many stress-induced genes by sequencing the transcriptome of sheepgrass [27]. In our previous studies, we reported the functions of several novel genes and TF genes in sheepgrass [31–33]. For example, *LcSAIN1*, *LcSAIN2*, and *LcFIN2* were identified as novel genes from sheepgrass and were shown to enhance salt and cold stress tolerance in transgenic Arabidopsis and rice [28, 29, 34]. The ectopic expression of the R1-MYB transcription factor gene *LcMYB1* from sheepgrass confers salt tolerance in transgenic Arabidopsis [35], and the overexpression of a novel cold-responsive transcript factor *LcFIN1* improved freezing tolerance in transgenic Arabidopsis [30].

Here, we report for the first time that an unknown function MYB transcription factor gene, *LcMYB4*, from sheepgrass is involved in the cold stress response. Our results indicated that *LcMYB4* plays an important positive role in transgenic Arabidopsis under chilling and freezing tolerance, and we are the first to find that the LcMYB4 protein can interact with the LcFBA1 protein in sheepgrass.

## Results

### Molecular characterization analysis of *LcMYB4* and homologous genes

**Fig. 1:**
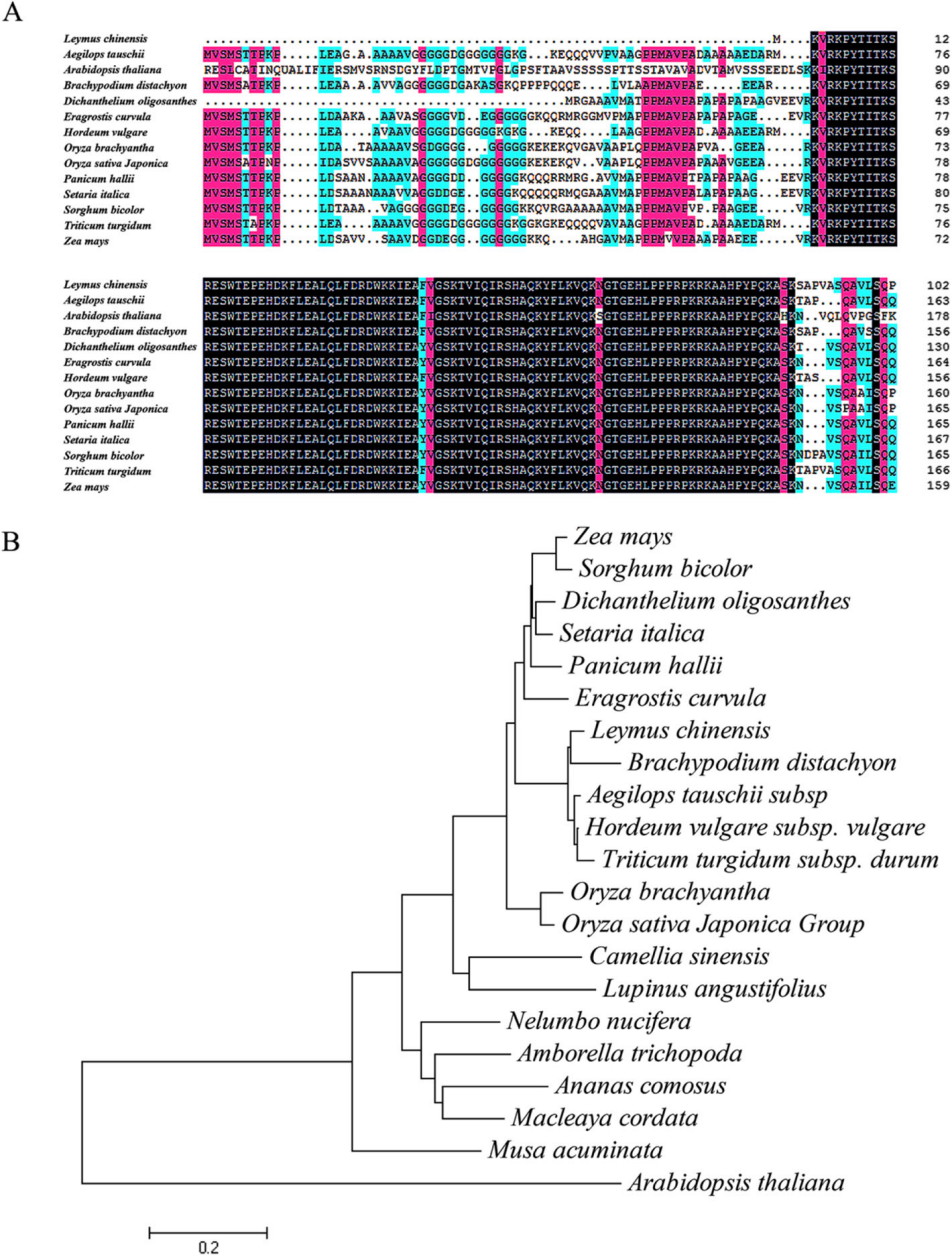
Sequence analysis of LcMYB4 and its homologs. **a** Comparison of the amino acid sequences of thirteen MYB4 proteins, i.e., *Arabidopsis thaliana*, *Aegilops tauschii*, *Brachypodium distachyon*, *Dichanthelium oligosanthes*, *Eragrostis curvula*, *Hordeum vulgare*, *Oryza brachyantha*, *Oryza sativa Japonica*, *Panicum hallii*, *Setaria italic*, *Sorghum bicolor*, *Triticum turgidum*, *Zea mays*, etc. **b** Phylogenetic analysis of LcMYB4 and homologs was constructed based on peptide sequences using a neighbor–joining method

Here, a MYB TF gene, *LcMYB4,* from sheepgrass was cloned. The ORF of this gene is 828 bp and encodes a predicted protein of 275 amino acids with a predicted molecular mass of 30.56 kDa, and an isoelectric point of 5.99 (GenBank accession no: MN327641). The SignalP 4.0 and TMHMM 2.0 server predicted showed that there was no signal peptide and transmembrane helix in the protein. Multiple sequence alignment analysis showed that the LcMYB4 protein had high similarity with 11 MYB homologous proteins in *Hordeum vulgare*, *Aegilops tauschii*, *Triticum turgidum*, *Zea mays*, *Oryza sativa*, *Brachypodium*, *Arabidopsis thaliana*, etc., and all the proteins had SANT/MYB superfamily domains (Fig. 1a). The homology of LcMYB4 with *H. vulgare* was 95% (Accession: BAJ91904.1), with *A. tauschii* was 94% (Accession: XP_020178881.1), with *T. turgidum* was 94% (VAI80123.1), with rice was 80%(XP_015643724.1) and with Arabidopsis was 58% (NP_568776.2) (Fig. 1b). However, the functions of *LcMYB4* genes and their homologues have not been verified.

### Expression analysis of *LcMYB4*

**Fig. 2:**
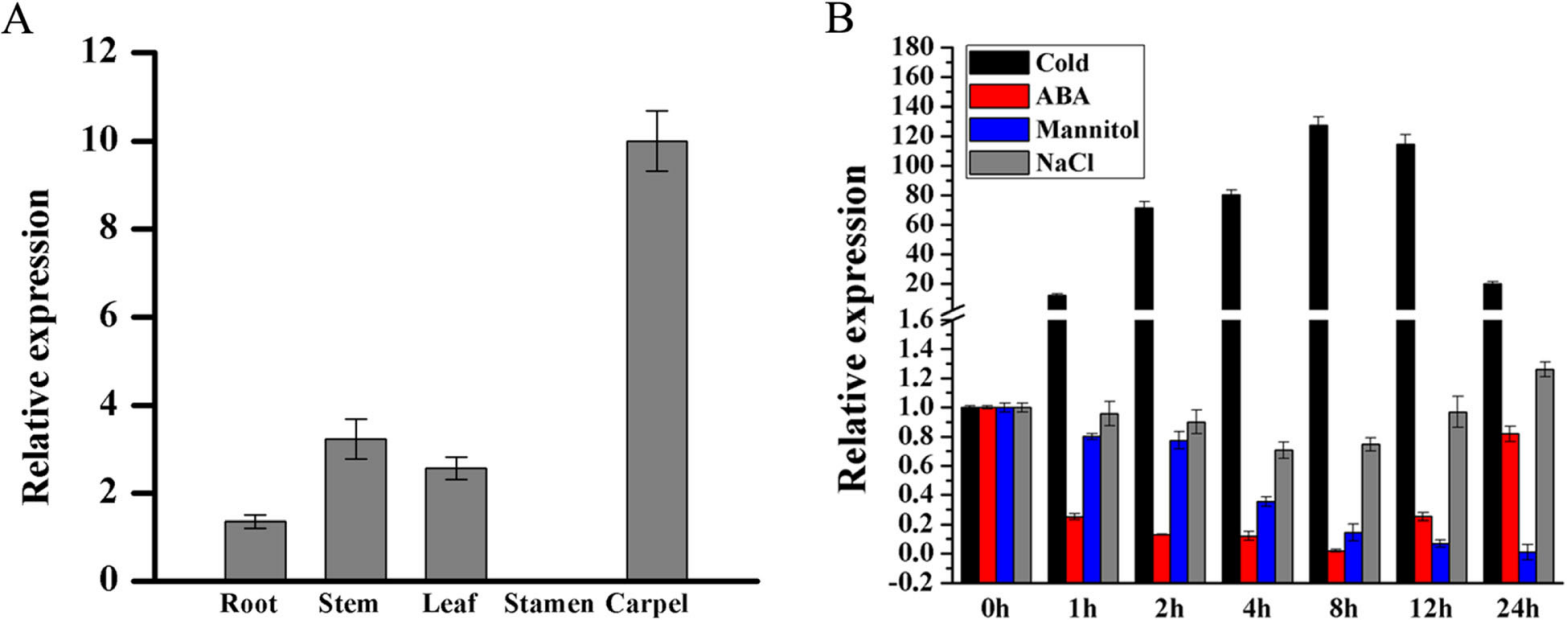
The expression pattern analysis of *LcMYB4*. **a** Expression of *LcMYB4* in various tissues as determined by quantitative RT-PCR. **b** Four-week-old seedlings were treatment with different stress and the expression of *LcMYB4* under cold, ABA mannitol, and NaCl treatments for the indicated lengths of time as determined by quantitative RT-PCR. Data are presented as the mean of three replicates ± SD

The expression of the *LcMYB4* gene in leaves, stems, roots, stamens, and carpel was analyzed by quantitative RT-PCR, and the results showed that *LcMYB4* was more highly expressed in carpel, stem, and leaf, but the relative expression in stamens was lower (Fig. 2a). Moreover, the expression pattern of *LcMYB4* under abiotic stress was also studied. At 4 weeks, seedlings were subjected to low temperature at 4 °C, 100 μM abscisic acid (ABA), 200 mM NaCl, or 300 mM mannitol. RNA was extracted at different time points (0 h, 1 h, 2 h, 4 h, 8 h, 12 h and 24 h). QRT-PCR analysis showed that the level of the *LcMYB4* gene was significantly induced by cold treatment, with the highest expression at 8 h. However, *LcMYB4* gene expression was not induced by NaCl treatment and was inhibited by ABA and mannitol treatment (Fig. 2b).

### Subcellular localization and transactivation assay of LcMYB4

**Fig. 3:**
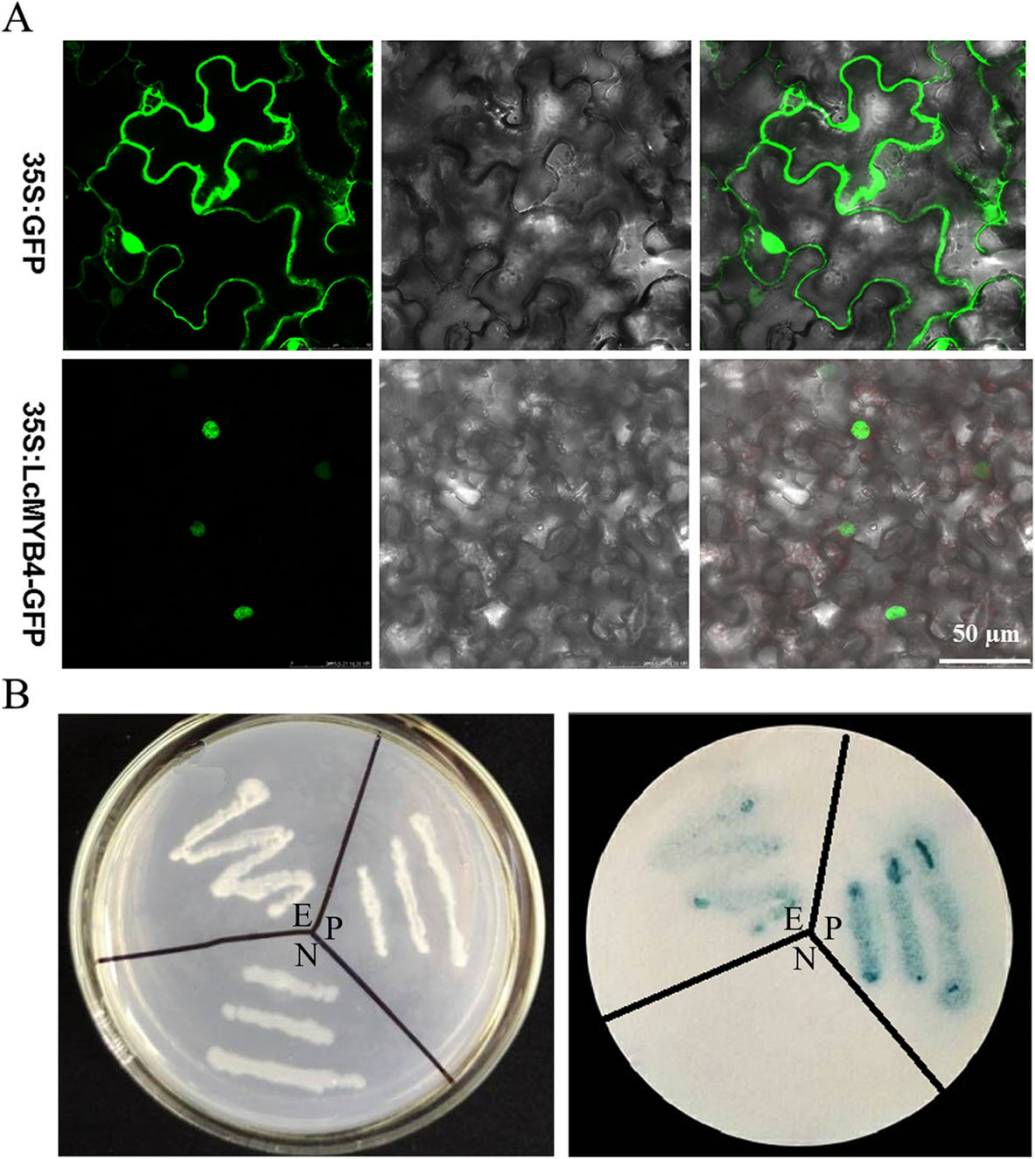
Subcellular localization and transactivation assay of LcMYB4. **a** Subcellular localization of LcMYB4 in tobacco (*Nicotiana benthamiana*) leaf epidermal cells. 35S:GFP and 35S:LcMYB4 plasmids were transformed into tobacco leaf epidermal cells, and GFP fluorescence was visualized under a confocal laser-scanning microscope. From left to right, GFP fluorescence, bright-field, and overlays of the GFP fluorescence and bright-field. Bar = 50 μm. **b** Transactivation assay of LcMYB4. E, Experimental group (pBridge-*LcMYB4*) P, Positive control (pBridge-*LcWRKY5*); N, Negative control (pBridge)

To determine the subcellular localization of LcMYB4, the gene was loaded into the pCAMBIA1302 vector, and the fused protein, 35S::LcMYB4-GFP, was immediately transformed into tobacco (*Nicotiana benthamiana*) leaf epidermal cells. The results showed that the fluorescence of 35S::GFP was distributed in the nucleus, cell membrane and cytoplasm, while that of 35S::LcMYB4-GFP was only located in the nucleus (Fig. 3a).

The ORF of LcMYB4 was inserted into the pBridge vector, and the recombinant vector pBridge-LcMYB4 (experimental group), empty vector pBridge (negative control) and pBridge-LcWRKY5 (positive control) were each transferred into yeast strain AH109. The yeast was screened and cultured on SD/−His-Trp-free double-deficiency medium, and then the activity of beta-galactosidase was detected. As shown in Fig. 3b, the yeast strains with empty vectors could not grow on double-deficiency medium, while the yeast strains with pBridge-LcMYB4 and pBridge-LcWRKY5 could grow on SD/−His-Trp medium. Furthermore, the filter paper of the positive control (pBridge-LcWRKY5) and the experimental group (pBridge-LcMYB4) showed obvious blue, while the negative control (pBridge) did not show blue (Fig. 3b). These results suggested that the LcMYB4 protein had transcriptional activation activity.

### Overexpression of *LcMYB4* enhances chilling resistance during seed germination

**Fig. 4:**
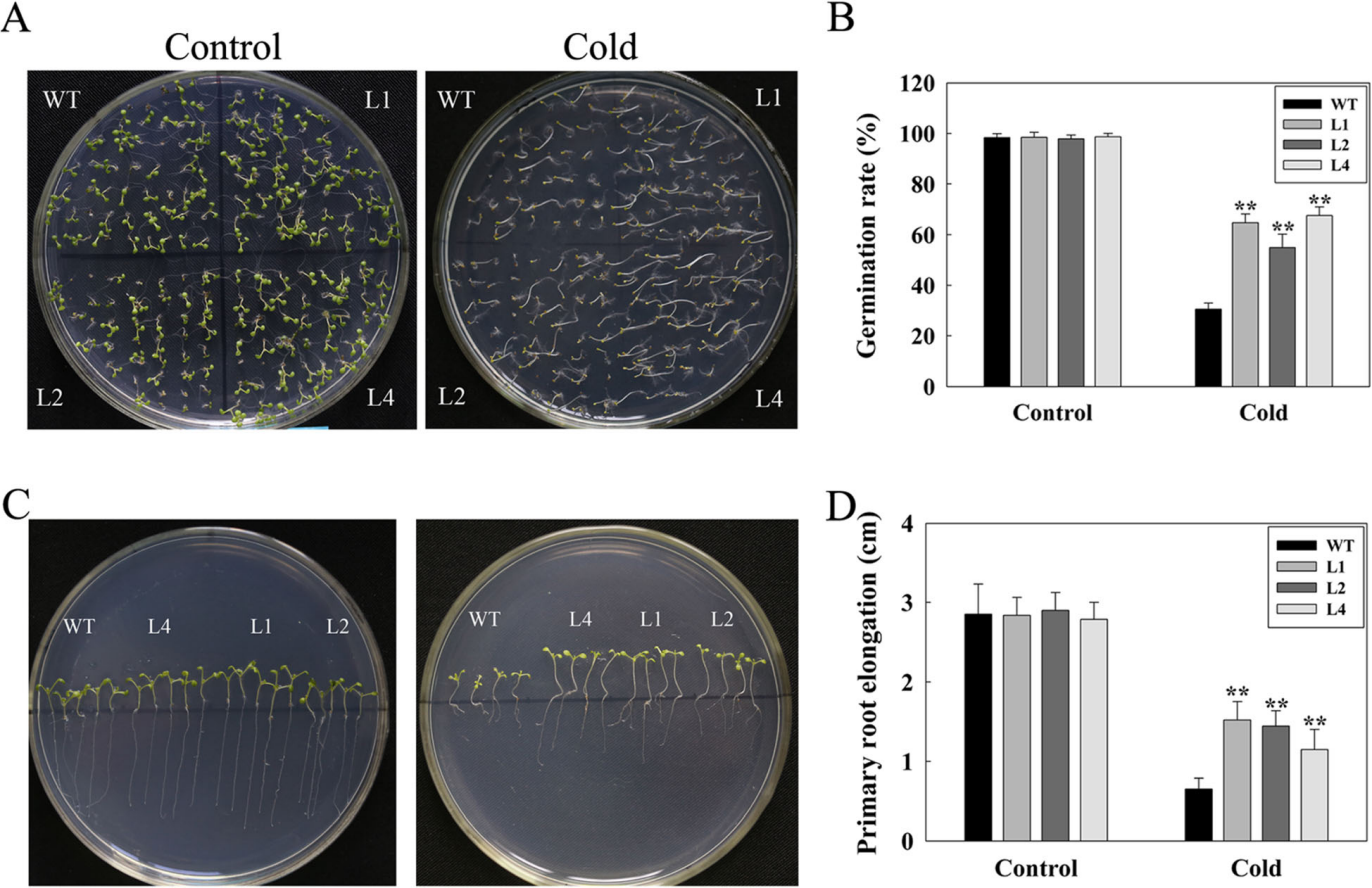
Seed germination and seedling development of *LcMYB4*-overexpression and WT Arabidopsis plants under chilling stress. **a** WT plants and three *LcMYB4*-overexpression lines were germinated on 1/2 MS medium with or without chilling stress (control and cold). **b** The seed germination rate of the *LcMYB4*-overexpression lines and WT plants under control for 7 d and 4 °C stress for 50 d. (**c**, **d**) Root development of overexpression and WT plants with or without cold stress. The primary root elongation was measured 50 d under 4 °C treatment. Experiments were performed in triplicate, and ** is indicated *P* < 0.01

Since *LcMYB4* was significantly induced by cold treatment, we further tested whether *LcMYB4* is involved in the adaptation to chilling stress during the seed germination stage using three overexpressing lines, L1, L2 and L4, and the transcription levels are shown in Additional file 2: Figure S1. The results indicated that there were no differences in the germination rates of overexpressing lines and WT plants under normal condition, while the germination rates of overexpressing lines were 64.3, 54.9, and 69.1%, respectively, and WT was only 30.1% under 4 °C conditions (Fig. 4a, b). In addition, the effect of chilling stress on seedling development ware also investigated. Our results indicated that the growth of shoot or root were no significant difference between overexpressed plants and wild plants (Fig. 4c). However, the primary root elongation of overexpressing plants was significantly longer than the WT plants at 4 °C for 50 d (Fig. 4c, d). These results indicate that *LcMYB4* overexpression enhances chilling resistance during seed germination and seedling development.

### Effects of *LcMYB4* overexpression on freezing tolerance

**Fig. 5:**
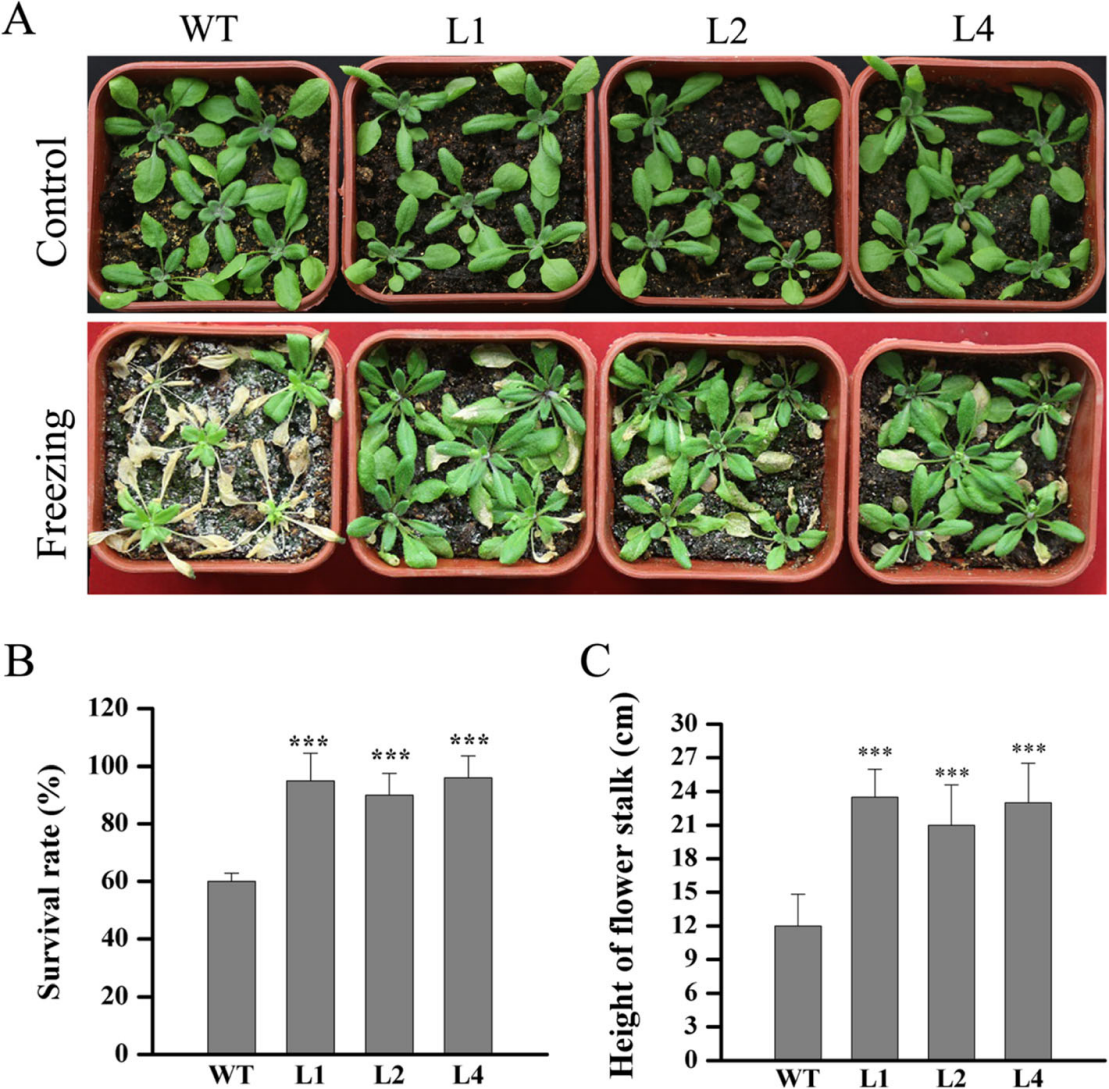
Freezing tolerances of *LcMYB4*-overexpressing Arabidopsis. **a** Performance of WT and transgenic lines under normal conditions (control) and freezing treatment (− 8 °C for 8 h, and recovered for 10 d). (**b**, **c**) Survival rates and height of flower stalk of transgenic and WT Arabidopsis after freezing treatments. Three-old plants were grown at 4 °C for 2 d, transferred to − 8 °C for 8 h, and then grown under normal conditions to recovery for 10 d. Experiments were performed in triplicate, and *** is indicated *P* < 0.001

We next investigated whether *LcMYB4* is involved in the adaptation to freezing stress in transgenic Arabidopsis. Three weeks of WT and *LcMYB4*-overexpressing plants were acclimated at 4 °C for 2 d, − 8 °C freezing treatment for 8 h, and then recovered for 10 d. Our results showed that there were no striking phenotypic differences between *LcMYB4*-overexpressing lines and WT plants under normal growth conditions; however, the overexpressing lines displayed more freezing tolerance than WT plants after − 8 °C freezing treatment (Fig. 5a). The survival rates of three overexpressing lines were 95, 92, and 96%, while that of wild type was only 60% (Fig. 5b). In addition, the pedicel length of the transgenic plants was significantly longer than that of the wild type (Fig. 5c). These results indicated that the overexpression of *LcMYB4* significantly improved the freezing resistance of Arabidopsis.

### *LcMYB4* overexpression enhanced chilling stress resistance during the reproductive stage

**Fig. 6:**
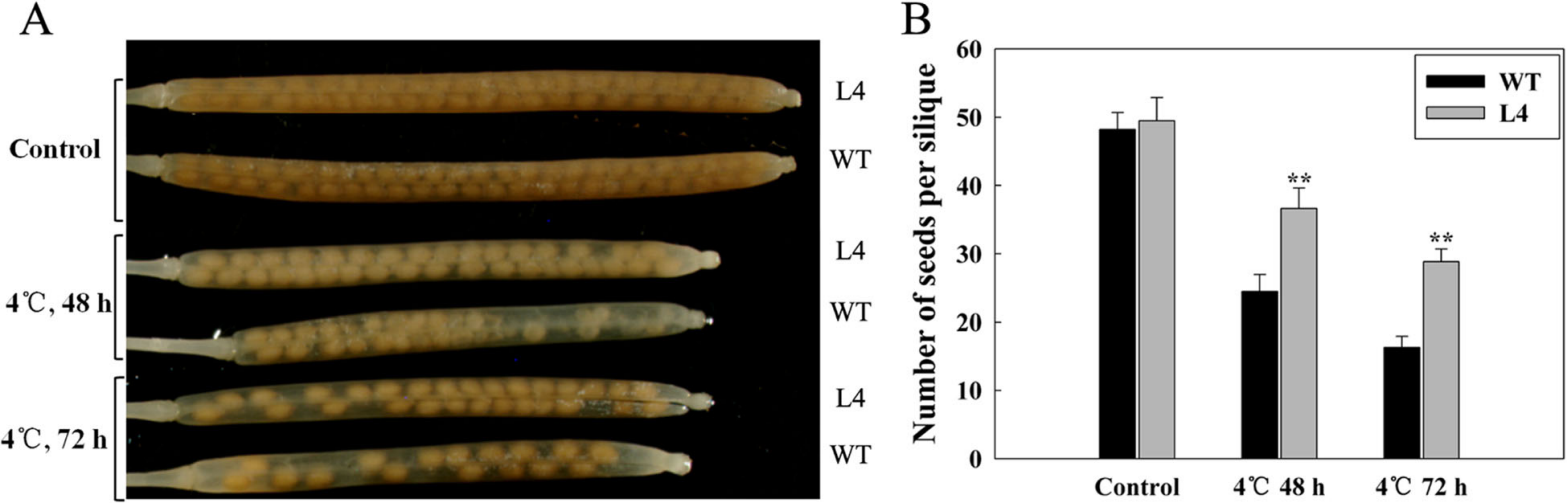
Chilling tolerances of *LcMYB4*-overexpressing Arabidopsis during the reproductive stage. **a** Phenotype of seed set within mature siliques from chilling-stressed transgenic and wild-type Arabidopsis. **b** The number of seeds per silique of cold-stressed transgenic and wild-type Arabidopsis. Experiments were performed in triplicate, and ** is indicated *P* < 0.01

To observe the effect of chilling on the reproduction stage of transgenic Arabidopsis, we selected L4 to further study which transcription levels were highest than other transgenic lines (Additional file 2: Figure S1). Then, we cut off the flowering florets of WT and L4 plants, leaving the florets to be pollinated during the florescence stage. WT and transgenic plants were treated in a low temperature incubator at 4 °C for 0 h, 48 h and 72 h. Then, they were moved back to the normal condition at 22 °C. After the end of seed filling, at least 30 long-horned fruits were sampled and calculated for each silique. Under normal growth conditions, the average number of seeds per mature silique was approximately 48 (Fig. 6 a, B). Under chilling stress for 48 h, the number of seeds in WT decreased significantly, with an average seed number of 23 per siliques, but only slightly in L4 seeds. Under chilling stress for 72 h, the average number of seeds in WT and L4 siliques was 16 and 28, respectively (Fig. 6b). This indicated that the fertility of *LcMYB4* transgenic Arabidopsis was significantly higher than that of wild type plants under chilling treatment.

### *LcMYB4* overexpression increased cold stress response-linked physiological indices

**Fig. 7:**
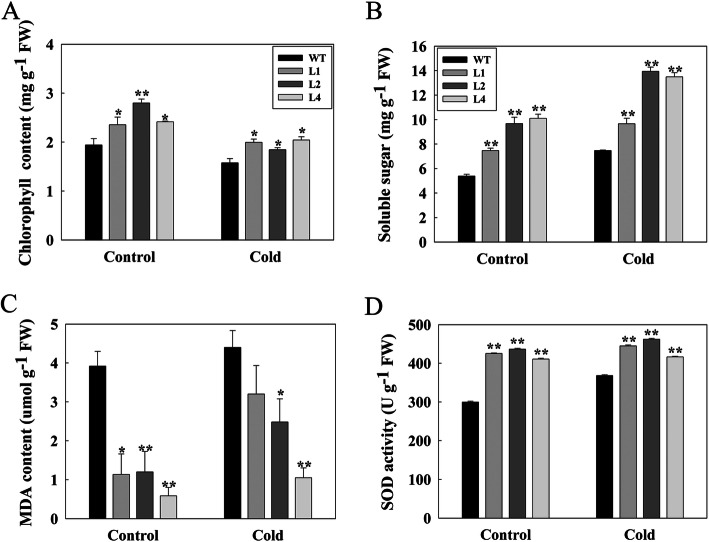
Analysis of chlorophyll (**a**), soluble sugar (**b**), MDA (**c**), and SOD (**d**) contents in *LcMYB4*-overexpressing lines and WT plants. Three-week-old plants were transferred to 4 °C for 2 d, and the leaves were collected and measured. Experiments were performed in triplicate. * and ** are indicated *P* < 0.05 and *P* < 0.01, respectively

Some physiological indices related to cold stress in Arabidopsis were determined. Chlorophyll is an important indicator of plant response to cold stress [36]. Our results showed that the total chlorophyll content of transgenic Arabidopsis was significantly higher than that of wild type plants (Fig. 3a), suggesting that chlorophyll degradation in transgenic plants may be less in transgenic plants under stress. Under normal conditions, the soluble sugar content of transgenic plants was significantly higher than that of wild type plants. Soluble sugar, as an important osmoprotectant in cell structure, can prevent plant cell dehydration and improve plant cold tolerance [6, 37]. The increase in soluble sugar content indicates that transgenic plants have cold tolerance potential. Moreover, the soluble sugar content in transgenic lines is significantly higher than that in WT under cold conditions (Fig. 7b), which implies that the tolerance to low cold stress can be improved by increasing the soluble sugar content by overexpressing the *LcMYB4* gene (*P* < 0.01).

We also measured malondialdehyde (MDA), which is one of the products of peroxidized polyunsaturated fatty acids in plant membranes, and the higher content of MDA, the higher membrane damage caused by abiotic stress to plant cells [38]. Under cold conditions, the MDA content in one of the three *LcMYB4*-overexpression lines was less severe than that in wild-type plants, indicating a higher degree of lipid peroxidation in the transgenic plants (Fig. 7c). Superoxide dismutase (SOD), as a major antioxidant enzyme, can scavenge oxygen free radicals in plants and enhance their environmental adaptability [30, 39]. Furthermore, we analyzed the activities of SOD in *LcMYB4*-overexpressing lines and WT plants under low temperature (4 °C) and normal growth (22 °C) conditions. The results showed that the SOD activity in the L1, L2 and L4 transgenic lines was higher than that in WT plants under both normal and cold stress conditions (*P* < 0.01), suggesting that the antioxidant ability of transgenic plants was improved and that the ability to resist cold was enhanced (Fig. 7d). Altogether, these observations suggest that the overexpression of *LcMYB4* in Arabidopsis improved the plant’s tolerance to cold stresses.

### *LcMYB4*-overexpressing plants show altered expression of cold-responsive genes

**Fig. 8:**
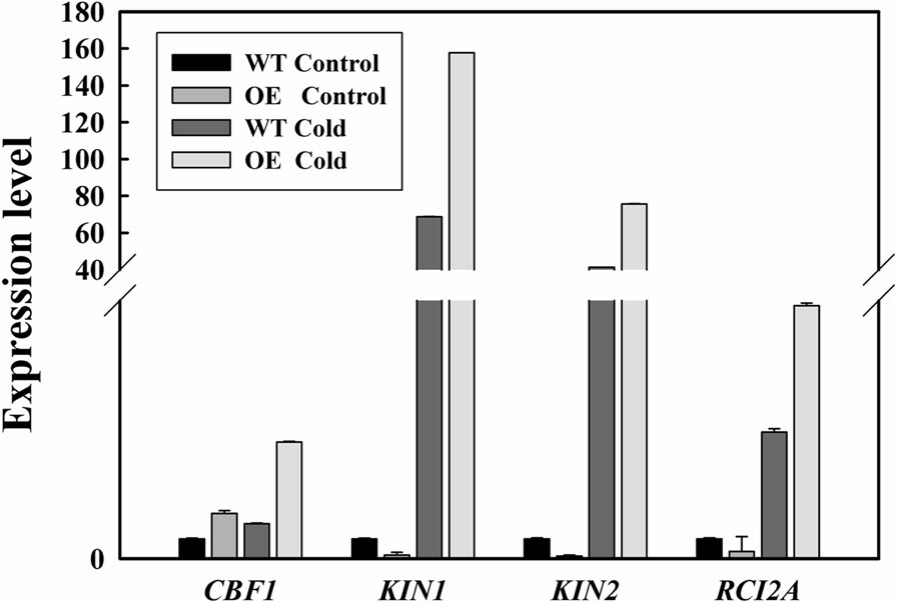
Expression levels of four cold-inducible genes in WT and LcMYB4-overexpressing Arabidopsis with or without cold stress. Total RNA was extracted from the 14-d-old seedlings under control and cold stress (4 °C) conditions for 24 h

We further studied the relative expression levels of four cold stress-responsive genes to elucidate possible stress response mechanisms in the transgenic lines and WT plants, with and without cold stress. Our results suggested that the expression of the transcription factor genes *CBF1* and the cold-inducible genes *KIN1*, *KIN2* and *RCI2A* were significantly increased in transgenic plants with cold treatment compared with the control plants (Fig. 8, Additional file 3: Figure S2). However, the expression levels of these genes were not significantly different between transgenic plants and WT plants without cold stress.

### Identification and analysis of LcMYB4-interacting proteins

To identify proteins that interact with LcMYB4, the cDNA was cloned into a pET30a expression vector. To obtain the purified recombinant protein, LcMYB4 was fused with a His tag, and the recombinant plasmid was induced to be expressed in *E. coli* BL21 (DE3) and purified by His column. An IPTG-induced band of the expected size of 40 kDa (with the theoretical value of LcMYB4 protein 31 kDa plus the 6 His tag) and the purified LcMYB4-His recombinant protein is shown in Additional file 4: Figure S3.

Furthermore, the protein extracts of sheepgrass seedlings were added, and the nonspecific binding proteins were removed by using imidazole buffer (10 mM–100 mM) with different concentration gradients. After the LcMYB4 binding protein was eluted with 250 mM imidazole buffer, the differential bands were detected by SDS-PAGE electrophoresis and identified by mass spectrometry. The results of mass spectrometry identification are in Additional file 5: Table S2. Based on sequence alignment and homologous gene function prediction, we identified a candidate protein for LcMYB4 by mass spectrometry, which is a fructose-1,6-bisphosphate aldolase (FBA1). The ORF region of the gene was cloned and named *LcFBA1*. The LcFBA1 protein has 98, 97, 94 and 88% homology with fructose-1,6-diphosphate aldolase 1 in *Triticum aestivum*, *Aegilops tauschii*, *Brachypodium distachyon*, and *Oryza sativa*, respectively (Additional file 6: Figure S4).

**Fig. 9:**
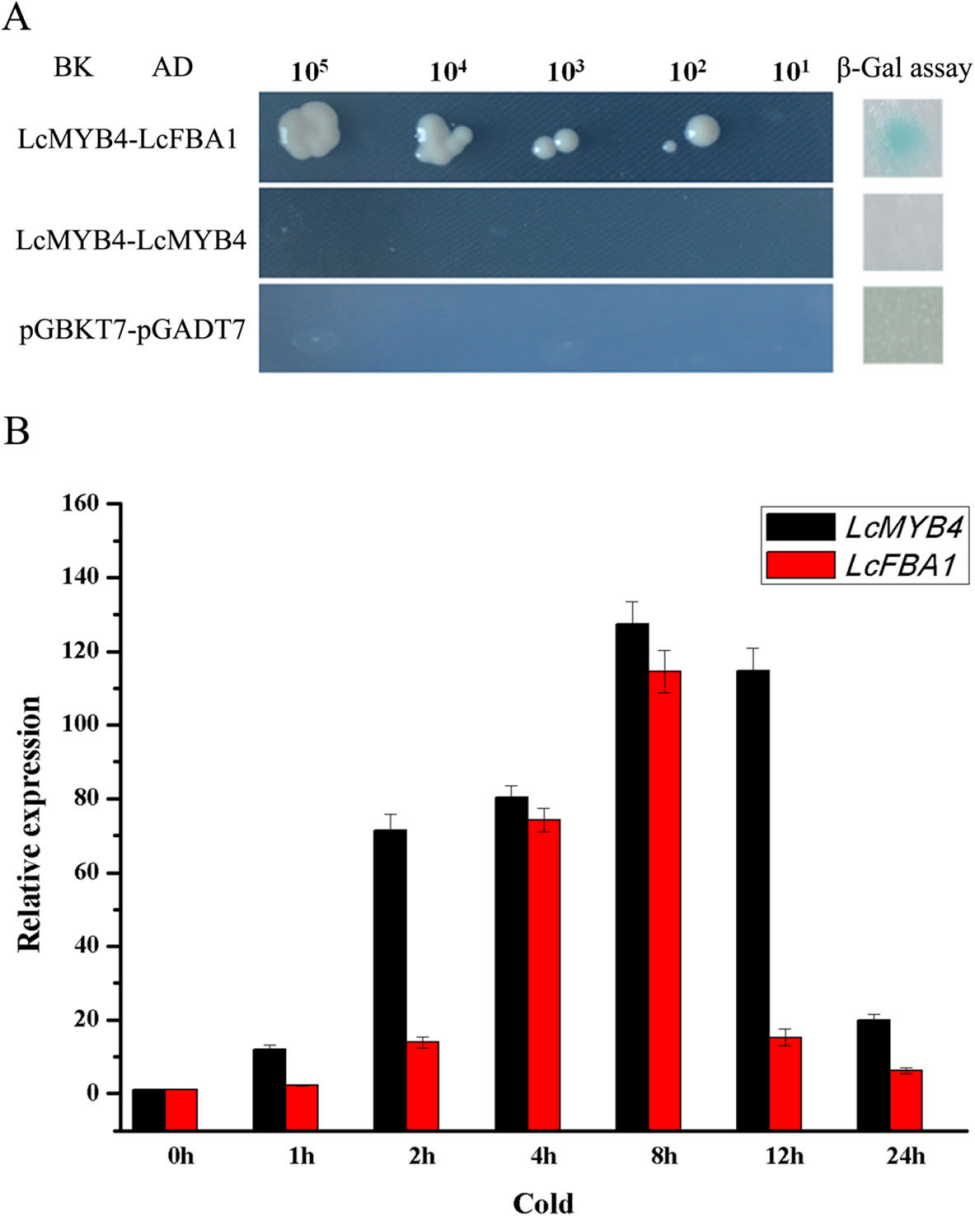
Analysis of LcMYB4-interacting protein. **a** Yeast two-hybrid analysis of LcMYB4 and LcFBA1. Serial dilutions (10^5^–10^1^) of yeast cells containing different plasmid combinations were grown on the selective medium plates SD/−Trp-Leu-His-Ade/5 mM-AT. Colony growth indicated that the interaction represents stronger interactions. The interaction of pGADT7 and pGBKT7 served as a negative control. **b** Expression pattern analysis of *LcMYB4* and *LcFBA1* under cold stress by quantitative RT-PCR. Data are presented as the means of three replicates ± SD

*LcFBA1* and *LcMYB4* were then linked to AD and BD vectors of the yeast two-hybrid system, respectively. To avoid false positive phenomena caused by self-activation, we first transfected the LcMYB4 gene into a BD yeast expression vector to eliminate self-activation. The results showed that yeast with BD vector could not grow on SD-Trp-His-Ade + Amp, 5 mM 3-AT. The yeast transformed with AD and BD plasmids could grow normally on SD-Leu-Trp-His-Ade + Amp + Kan, 5 mM 3-AT medium. The interaction between pGADT7 and the pGBKT7 empty vector served as a negative control. In the middle, the yeast medium containing two plasmids was diluted to different multiples and cultured on four-deficiency medium with 5 μL. After 1 week, yeast growth was observed. The right side corresponds to the detection of beta-galactosidase activity. The results showed that the LcMYB4 protein interacted with the LcFBA1 protein, as shown in Fig. 9a.

The expression of *LcFBA1* and *LcMYB4* was significantly induced by low temperature. The expression of LcFBA1 was upregulated by 13.94 times that of CK at 2 h of cold treatment and reached its peak at 8 h of cold treatment, which was 114 times that of CK (Fig. 9b). The expression of the LcMYB4 gene peaked at 8 h under low temperature stress (Fig. 9b).

## Discussion

### *LcMYB4*, a MYB transcription factor gene, induced by cold stress from sheepgrass

Sheepgrass is one of most important perennial forages in China and can survive severe environmental conditions. Through transcriptome analysis, many TF genes were found to be significantly induced by abiotic stress in sheepgrass, such as *LcDREB2a* [40], *LcWRKY5* [32], and *LcMYB1* [35]. Meanwhile, the function of several cold-related genes has been reported in our previous studies [28, 30]. Here, we first reported the mechanisms and functional roles of *LcMYB4*, from sheepgrass. Our results indicated that the genes contains the SANT/MYB superfamily domain and that the homology of LcMYB4 with *H. vulgare*, *A. tauschii*, and rice were 95, 94, 80%, respectively (Fig. 1). However, the function of its homologous genes has also not been studied.

Moreover, the expression of *LcMYB4* was rapidly induced by cold treatment; its expression peaked after exposure to 4 °C for 8 h and decreased thereafter (Fig. 2). However, the expression of *LcMYB4* was not upregulated by salt and ABA stresses. Previous studies found that the many *MYB* genes from rice and a sheepgrass R1-MYB TF *LcMYB1* are also induced by cold stress [5, 25, 35, 41, 42]. In addition, subcellular localization and transcriptional activation experiments showed that LcMYB4 was located in the nucleus and had transcriptional activation activity (Fig. 3). Therefore, *LcMYB4* is MYB gene in sheepgrass that is induced exclusively by cold stress.

### *LcMYB4* positively modulates chilling and freezing tolerance in Arabidopsis

To investigate the role of *LcMYB4* in tolerance to chilling and freezing stress, this study generated transgenic Arabidopsis with *LcMYB4* overexpression because genetic transformation in sheepgrass is still very difficult [43]. *LcMYB4* overexpression enhanced chilling resistance during seed germination and seedling development, mainly reflected on the germination rates, and primary root elongation was significantly higher in transgenic plants than in WT plants (Fig. 4). Furthermore, *LcMYB4* overexpression enhanced freezing tolerance after 2 d of cold acclimation in transgenic Arabidopsis (Fig. 5).

In addition, the effect of chilling on the seed setting of Arabidopsis was studied, and the results indicated that the number of seeds per silique in the WT line was less than in the transgenic line under chilling stress for 48 h (Fig. 6). Several studies have found that the heterologous expression of the MYB gene can enhance the abiotic stress resistance of transgenic *A. thaliana* [44, 45]. For example, a wheat TaMYB30-B can improve drought stress tolerance in transgenic Arabidopsis [46]. The overexpression of the rice *Osmyb4* and *OsMYB3R-2* genes increases the freezing tolerance of transgenic *A. thaliana* plants [23, 41]. Moreover, recent research found that the Myb-like protein DRMY1 regulates cell expansion and seed production in Arabidopsis [19], while our research indicates that *LcMYB4* overexpression increases the number of seeds per silique under chilling stress. Thus, our results clearly reveal that *LcMYB4* is an important positive mediator in the response to chilling and freezing stress.

### Possible mechanisms responsible for the enhanced plant cold resistance in *LcMYB4*-overexpressing plants

To elucidate the possible mechanisms for the increased cold tolerance in *LcMYB4*-overexpressing plants, several physiological indices were analyzed, such as chlorophyll, soluble sugar, MDA and SOD. Chlorophyll content has been widely used to evaluate the plant stress response [28], and our results indicated that the total chlorophyll content of transgenic plants was higher than that of WT plants (Fig. 7Ab). Previous studies showed that the soluble sugar contents increased significantly when the plant responded to different environmental stresses [5, 47, 48]. Our study found that expressing *LcMYB4* increased the biosynthesis of soluble sugars in transgenic plants under normal and cold conditions (Fig. 7b), which may be partially responsible for the increased cold tolerance in *LcMYB4*-overexpressing plants.

Furthermore, this study indicated that MDA content and SOD activity were also significantly different between transgenic plants and wild-type plants. MDA is an indicator of plant cell membrane damage under stress [38], and SOD can scavenge oxygen free radicals in plants and enhance their environmental adaptability [30, 39]. The MDA content in one of the three *LcMYB4*-overexpression lines was less severe than that in WT plants (Fig. 7c), while the activities of SOD in *LcMYB4*-overexpression lines was higher than that of WT plants (Fig. 7d). In this study, we speculate that it may be due to the alleviation of oxidative damage and membrane damage, resulting in the enhanced cold resistance of *LcMYB4*-overexpressing plants.

### Overexpression of *LcMYB4* increases the expression of cold-responsive genes in Arabidopsis

To clarify the function of genes, we should further understand the abiotic stress signaling pathways in transgenic plants. CBF pathways are the most important pathways in the mediation of cold response and involve three TFs, *CBF1*, *CBF2* and *CBF3* [9, 49, 50]. Our results of four cold-response gene expression analyses suggest that the *CBF1* genes are strongly expressed under cold stress in transgenic plants compared with WT plants. Previous studies indicated that an inhibitor of calcium-dependent protein kinases (CDPKs) and calmodulin inhibited the *KIN* genes expression and prevented cold acclimation [51]. *KIN1* and *KIN2* are two cold-induced genes in Arabidopsis that are coordinately regulated in response to cold stress [51]. Furthermore, *RCI2A* (rare cold inducible gene 2A) is also an important cold-inducible gene in Arabidopsis [52]. We detected the expression levels of the cold-inducible *KIN1*, *KIN2*, and *RCI2A* genes in transgenic and WT Arabidopsis. The expression levels of the *KIN1* and *KIN2* genes were significantly increased under cold conditions in transgenic plants (Fig. 8). Similar to the *KIN* genes, the overexpression of *LcMYB4* significantly upregulated the expression level of the *RCI2A* gene. Overall, our results suggested that *LcMYB4* is a TF gene and that overexpression of the *LcMYB4* gene may involve cross-talk with the CBF1 TF gene and increase the expression of cold-inducible genes.

### LcMYB4 can interact with LcFBA1 protein

Fructose-1,6-bisphosphate aldolases (FBA) are one of the most important enzymes in the Calvin–Benson cycle (CBC), and *FBA* genes are significantly induced in response to various stresses; moreover, the overexpression of *FBA* genes can enhance the resistance of transgenic plants to abiotic stresses, including ABA, NaCl, low temperature and drought [53–55].. Previous study has found that FBA can bind to MADS proteins, which is a MADS box transcription factor, revealing possible important molecular functions in plant physiological and biochemical metabolism [56]. In the present study, we first found that LcFBA1 is a protein that interacts with LcMYB4 in sheepgrass (Additional file 5: Table S2**).** Furthermore, our results showed that LcMYB4 could interact with LcFBA1 in vitro, as verified by yeast two-hybrid assays (Fig. 9a). In addition, the expression pattern analysis showed that both the *LcMYB4* and *LcFBA1* genes were both upregulated during cold induction (Fig. 9b). Thus, we speculated that LcMYB4 can interact with LcFBA1 to regulate the expression of downstream genes in response to cold stress in sheepgrass.

## Conclusions

Our study identified an unknown function transcription factor gene, *LcMYB4,* from sheepgrass that plays important roles in the plant response to cold stress. The overexpression of *LcMYB4* in Arabidopsis led to greater germination rate, survival rate, and seed setting rate through increases in soluble sugar content, leaf chlorophyll content and SOD activity, upregulation of the expression level of *CBF1*, *KIN1*, *KIN2*, and *RCI2A* genes, and decreased MDA content under cold stress conditions. Furthermore, our results indicated that LcMYB4 can interact with the LcFBA1 protein in sheepgrass and that the expression of the *LcFBA1* gene was induced by cold stress. Therefore, we speculated that *LcMYB4* is a positive regulator that mediates cold tolerance and provides a promising genetic resource for improving the cold tolerance of other crops.

## Methods

### Plant materials, growth conditions and experimental treatments

Sheepgrass (variety Zhongke No. 1), *Arabidopsis thaliana* (Columbia-0 ecotype) and tobacco (*Nicotiana benthamiana*) were used in this study. Zhongke No. 1 is an improved variety by our laboratory, and certificated by the National Grass Seed Certification Committee in China in May 2014. Arabidopsis seeds acquired from the Arabidopsis Biological Resource Center (https://abrc.osu.edu/). *N. benthamiana* seeds were obtained from Dr. Yunhai Li of the Institute of Genetics and Developmental Biology, the Chinese Academy of Science. Arabidopsis and tobacco seeds were planted in pots and grown in a greenhouse under approximately 23 °C and a 16 h day/8 h night cycle.

The seeds of sheepgrass were grown in a mixture of peat moss and vermiculite (2:1, v/v) at 28/16 °C under 16-h light/8-h dark. Four-week-old seedlings were used for various stresses treatment. For cold stress, the seedlings were treated at 4 °C in a chamber. To test ABA, drought and salt stresses tolerance, the seedlings were irrigated with 100 μM ABA, 300 mM mannitol and 200 mM NaCl, respectively [33]. The seedlings were collected at different time intervals (0, 4, 8 and 12 h) after abiotic stress treatments. Furthermore, the leaves, stems, roots, stamen and carpel were sampled at the time of flowering stage and stored at − 80 °C for tissue-specific expression analysis.

### RNA isolation, gene clone and phylogenetic analysis

Total RNA from sheepgrass seedlings and various tissues were isolated use a TRIzol kit (Invitrogen, Carlsbad, CA, USA) and treated with RNase-free DNase I (TaKaRa, Dalian, China) according to the manufacturer’s instructions. Then, the first-strand cDNA synthesis was performed with a PrimeScript® RT Reagent Kit (TaKaRa, Dalian, China).

Full-length sequence of *LcMYB4* and *LcFBA1* was amplified using the gene-specific primers supplied in Additional file 1: Table S1, and the amplification conditions were 95 °C for 4 min, followed by 32 cycles of 98 °C for 10 s, and 68 °C for 90 s. All the PCR products were purified, and an A tail was added. Then, the products were ligated into a pMD18-T vector (TaKaRa, Dalian, China) and sequenced.

The cloned gene sequences were searched and aligned using the National Center for Biotechnology Information (NCBI; https://www.ncbi.nlm.nih.gov/). DNAMAN was used for sequence alignment, and the neighbor–joining algorithm of MEGA6.0 was used for the phylogenetic tree analysis of MYB4 proteins [57]. SignalP4.1 was used for signal peptide prediction [58].

### Real-time RT-PCR analysis

QRT-PCR was use to analysis the expression levels of *LcMYB4* gene in various organs and under different abiotic stresses, and the expression levels of cold-inducible genes in *LcMYB4*-overexpressing and WT Arabidopsis. The cDNA template was amplified with a qRT-PCR system (Roche Light Cycler 480 II, Germany) using 40 cycles of 95 °C for 5 s and 60 °C for 20 s, and the data were quantified using the 2^-ΔΔCt^ methodas described by our previous studies [28, 31, 33]. All primers used in this study are listed in Additional file 1: Table S1. *LcACTIN* was used as the internal control.

### Subcellular localization and transactivation assay of LcMYB4

The ORF sequence of *LcMYB4* was inserted into the *Spe*I and *Bgl*II sites of vector pCAMBIA1302 to express fusion proteins with green fluorescence protein (GFP). The intact leaves of 4-week-old wild type (WT) tobacco (*Nicotiana tabacum*) plants were injected with *Agrobacterium tumefaciens* harboring the pCAMBIA1302 vector and the fused protein pCAMBIA1302-LcMYB4-GFP. Transgene-derived expression was monitored 2 to 3 d after infiltration by confocal microscopy on an Olympus FV1000MPE microscope (Olympus, Japan), and fluorophores (GFP) were excited using an argon laser at 488 nm [29].

To investigate the transcription activation activity, the ORFs were cloned into the pBridge vector by *EcoRI* and *SalI* sites and subsequently transformed into yeast strain AH109 as described by our previous studies [30, 31]. The PBridge vector was used as a negative control, and pBridge-LcWRKY5 was used as a positive control [32]. The yeast was screened and cultured on SD/−His-Trp-free double-deficiency medium, and the β-galactosidase (LacZ reporter) assay was performed on sterile filter paper according to a previous study [59].

### Plasmid construction and plant transformation

To induce the constitutive expression of *LcMYB4*, the ORF sequences were inserted into the pSN1301 vector under the control of the cauliflower mosaic virus 35S (CaMV 35S) promoter [60]. The recombinant plasmid pSN1301-LcMYB4 was introduced into *Agrobacterium* strain EHA105 using a freeze-thaw method [61] and further transformed into Arabidopsis using the floral dip method [62]. The seeds of transgenic plants were selected on 1/2 MS medium supplied with hygromycin (50 mg L^− 1^) for 1 week and the seedlings were confirmed by PCR using the gene-specific primers, and then Arabidopsis seeds of T3 generations were obtained.

### Chilling and freezing stress tolerance assays in transgenic Arabidopsis

The seeds of WT and three transgenic Arabidopsis lines (L1, L2 and L4) were germinated on MS medium and transferred to a 4 °C incubator. The control was placed in a 22 °C greenhouse. After 4–5 weeks of low temperature incubation, the germination rate and cotyledon number were counted. A total of 150 seeds from each transgenic line and the WT line were used and each experiment was replicated three times. To determine the effect of chilling stress on root growth, seeds were germinated and grown on vertically standing plates. After 50 d of growth, the length of the primary root was measured.

To detect the chilling tolerances of *LcMYB4*-overexpressing Arabidopsis during the reproductive stage, transgenic and WT plants were grown in a small pot in a greenhouse. Flowers at developmental stage 13 were marked at a fixed time, the petals can be seen between the sepals and continue to elongate rapidly and the stigma is already receptive and anthesis occurs at this stage, [63, 64]. For the cold treatment, the plants were placed in a 4 °C low temperature incubator for 0 h, 48 h or 72 h and then transferred back to 22 °C. After seed filling, more than 30 siliques were sampled, and the average number of seeds per silique was calculated.

For freezing stress tolerance experiments, three-week-old Arabidopsis plants grown under normal growth conditions were cold-acclimated at 4 °C for 2 d and transferred to a low-temperature chamber at − 8 °C for 8 h. The survival rates and height of the flower stalk of transgenic and WT Arabidopsis were detected after 10 d of recovery under normal conditions. Furthermore, forty seedlings from each transgenic line and the WT line were used for the freezing tolerance assay. Each experiment was replicated three times.

### Physiological index measurements

To measure physiological indices, two-week-old seedlings were subjected to 4 °C for 2 d. The chlorophyll content was measured as reported [29, 30]. In brief, the leaves were immersed in 95% ethanol at 22 °C overnigh in darkness. Total superoxide dismutase (SOD) activity was monitored by nitro-blue tetrazolium (NBT) reduction method [28, 65]. The soluble sugar and malondialdehyde (MDA) contents were measured based on the method described by previous studies [28, 34].

### Extraction of total protein from sheepgrass

The extraction of whole protein from plants was mainly based on a previous study [66]. The leaves of 4-week-old sheepgrass seedlings were weighed at 0.5 g, and 5 mL of extract buffer E (50 mM Tris-HCl (pH 7.5), 50 mM NaF, 1 mM PMSF, 1 mM DTT, 1 mM Na_3_VO_4_, and 5 mM MgCl_2_) was added to a mortar to grind and form a homogenate. The homogenate was transferred to a 1.5 mL centrifuge tube for 14,000 x g for 40 min, and the supernatant was passed through a Millex-GV filter with a porosity of 0.22 μm to obtain the plant whole protein [67, 68].

### Pull-down analysis and screening of interactive proteins

To obtain the protein interacting with LcMYB4, the ORF sequence of *LcMYB4* was cloned into the pET-30a expression vector (Novagen, Madison, WI) to produce His_6_ tag-fused LcMYB4. The LcMYB4-His_6_ fusion protein was coupled to amylose resin (New England Biolabs) according to the manufacturer’s instructions, and then the protein extracts of sheepgrass seedlings were incubated with LcMYB4-His_6_–amylose resin at 4 °C for 4 h. Subsequently, the nonspecific binding proteins were washed five times with imidazole buffer (10 mM–100 mM) with different concentration gradients. The bound proteins were eluted with SDS/PAGE sample buffer and resolved by SDS/PAGE followed by mass spectrometry [69, 70].

### Yeast 2-hybrid analysis

For analysis the interaction of LcMYB4 and LcFBA1, the ORFs of the two genes were constructed into pGADT7 and pGBKT7 vectors (Clontech, Mountain View, CA, USA) and the constructs were cotransformed into *Saccharomyces cerevisiae* strain AH109. The transformants of LcMYB4 and LcFBA1 proteins were plated on SD/−Trp-Leu-His-Ade medium containting 3 mM 3-amino-1,2,4-triazole (3-AT) and incubated at 30 °C for 4–6 d as our previously described [33]. Meanwhile, the single transformant with pGBKT7 was used for the autoactivation analysis as our previously reported [33].

### Statistical analysis

SPSS 21.0 software (IBM, Chicago) was used for statistical analysis, and Student’s t-test was used to compare different samples. The bars showed the mean and standard deviation (SD) of three independent experiments, and the sterisks represent significant differences. The differences between WT and *LcMYB4*-overexpressing plants were considered statistically significant if *p*-values were < 0.001, < 0.01 or < 0.05.

## Supplementary information

**Additional file 1: Table S1.** All primers used in this study.

**Additional file 2: Figure S1.** (A) The positive transgenic lines determined by PCR. (B) The transcription levels of *LcMYB4* gene in overexpressing lines.

**Additional file 3: Figure S2.** The expression levels of *CBF1* gene in transgenic and WT plant during different time periods.

**Additional file 4: Figure S3.** (A) SDS-PAGE results of prokaryotic expression. M: Marker; 1: Before induction; 2–7: 0.5 mM IPTG induced for 1 h, 2 h, 3 h, 4 h, 5 h, 6 h. (B) The purification of recombinant protein pET30a-LcMYB4. M: Marker; 1: Before induction; 2: After induction; 3–7: The purified target protein.

**Additional file 5: Table S2.** LcMYB4 binding proteins in sheegrass seedling

**Additional file 6: Figure S4.** Phylogentic tree analysis of LcFBA1 and its homologous proteins.

## Data Availability

The gene sequence is available in the NCBI gene database under the accession number MN327641 (https://www.ncbi.nlm.nih.gov/nuccore/MN327641). All data generated or analyzed during this study are included in this published article and its supplementary information files (Additional files 1, 2, 3, 4, 5 and 6).

## Abbreviations

ABA: Abscisic acid
BLAST: Basic local alignment search tool
CaMV: Cauliflower mosaic virus
CDS: Coding sequences
cDNA: Complementary DNA
GFP: Green fluorescent protein
GUS: ß-Glucuronidase
MDA: Malondialdehyde
NaCl: Sodium chloride
NCBI: The national center for biotechnology information
ORF: Open reading frame
qRT-PCR: Quantitative real-time polymerase chain reaction
SOD: Superoxide dismutase
WT: Wild type
