# Supplementary figures and images for: LcMYB4, an unknown function transcription factor gene from sheepgrass, as a positive regulator of chilling and freezing tolerance in transgenic Arabidopsis
Source: BMC Plant Biol. 2020 May 27;20:238. doi: 10.1186/s12870-020-02427-y (PMC7333390; doi:10.1186/s12870-020-02427-y)

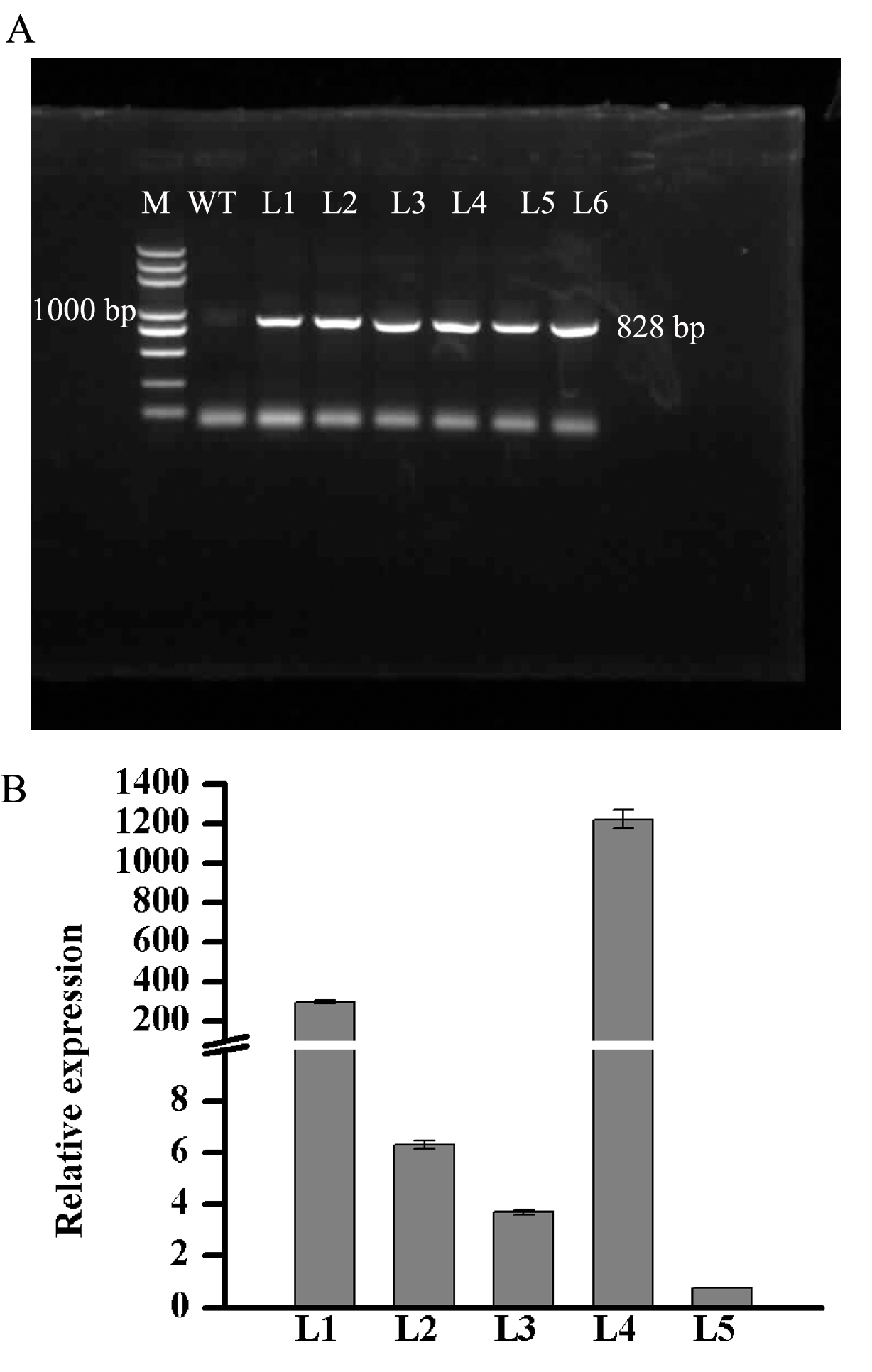

Supplement: Supplementary file 2 — Additional file 2: Figure S1. (A) The positive transgenic lines determined by PCR. (B) The transcription levels of LcMYB4 gene in overexpressing lines. [file 12870_2020_2427_MOESM2_ESM.tif]

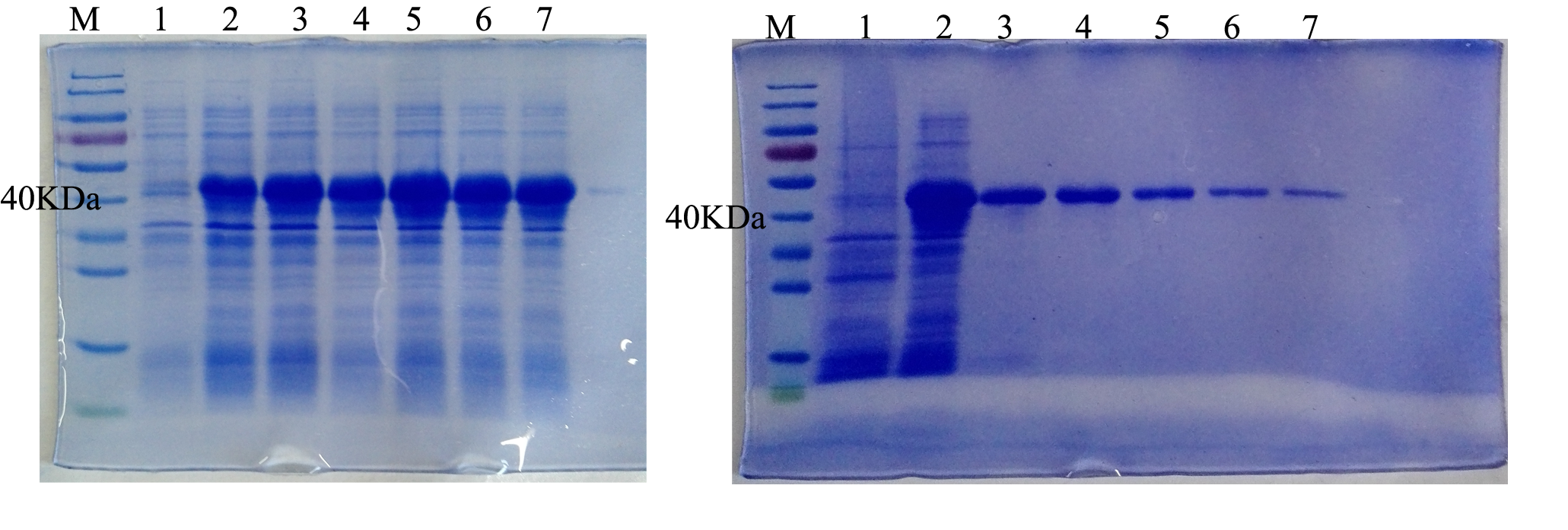

Supplement: Supplementary file 4 — Additional file 4: Figure S3. (A) SDS-PAGE results of prokaryotic expression. M: Marker; 1: Before induction; 2–7: 0.5 mM IPTG induced for 1 h, 2 h, 3 h, 4 h, 5 h, 6 h. (B) The purification of recombinant protein pET30a-LcMYB4. M: Marker; 1: Before induction; 2: After induction; 3–7: The purified target protein. [file 12870_2020_2427_MOESM4_ESM.tif]

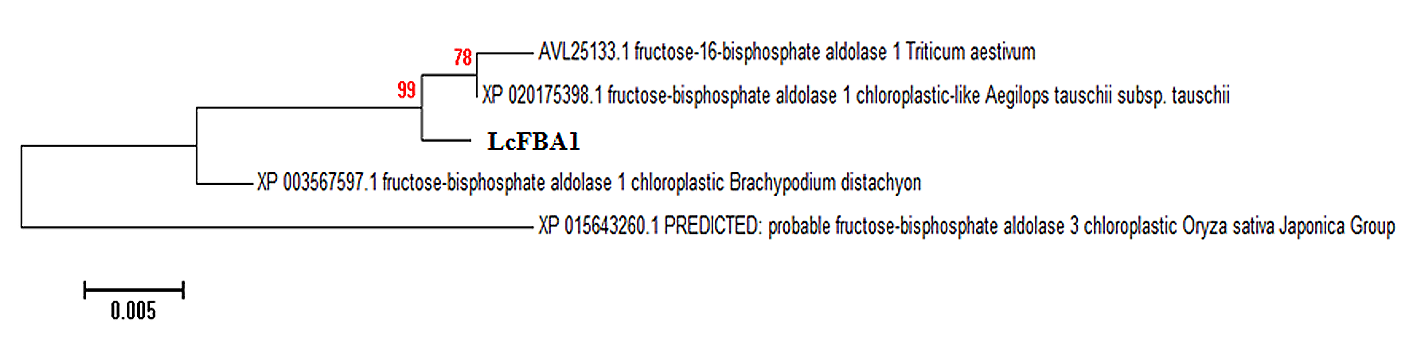

Supplement: Supplementary file 6 — Additional file 6: Figure S4. Phylogentic tree analysis of LcFBA1 and its homologous proteins. [file 12870_2020_2427_MOESM6_ESM.tif]
